# Supplementary material for: Consecutive deletions in a unique Uruguayan SARS-CoV-2 lineage evidence the genetic variability potential of accessory genes
Source: PLoS One. 2022 Feb 17;17(2):e0263563. doi: 10.1371/journal.pone.0263563 (PMC8853529; doi:10.1371/journal.pone.0263563)
Supplement: S1 Table — (PDF) [file pone.0263563.s001.pdf]

We gratefully acknowledge the following Authors from the Originating laboratories responsible for obtaining the specimens, as well as the Submitting laboratories where the genome data were generated and shared via GISAID, on which this research is based.

All Submitters of data may be contacted directly via www.gisaid.org

Authors are sorted alphabetically.

Accession ID	Originating Laboratory	Submitting Laboratory	Authors
EPI_ISL_750175	CENUR Este-Sede Rocha-UdelaR	Institut Pasteur de Montevideo	Ana Carolina Mendonça; Andrés Lizasoain; Camila Simoes; Cecilia Alonso; Cecilia Salazar; Daiana Mir; Fernando López-Tort; Fernando Motta; Gonzalo Bello; Ighor Arantes; Ignacio Ferrés; Jose Sotelo; Leticia Maya; Letícia Garay Martins; Luciana Appolinario; Lucía Spangenberg; Mailen Arleo; Mariana Brandes; Marilda Mendonça Siqueira; Marilda Tereza Mar da Rosa; María José Benitez-Galeano; Martín Graña; Matías Castells; Matías Victoria; Matías Salvo; Natalia Rego; Natalia Reyes; Pablo Smircich; Paola Cristina Resende; Rodney Colina; Tamara Fernandez-Calero; Tania Possi; Tatiana Schäffer Gregianini; Verónica Noya; Yasser Vega
EPI_ISL_751184, EPI_ISL_751185, EPI_ISL_751186, EPI_ISL_751187, EPI_ISL_751188, EPI_ISL_751189, EPI_ISL_751190			
see above	CENUR Litoral Norte – UdelaR, Salto, Uruguay	Institut Pasteur de Montevideo	Ana Carolina Mendonça; Andrés Lizasoain; Camila Simoes; Cecilia Alonso; Cecilia Salazar; Daiana Mir; Fernando López-Tort; Fernando Motta; Gonzalo Bello; Ighor Arantes; Ignacio Ferrés; Jose Sotelo; Leticia Maya; Letícia Garay Martins; Luciana Appolinario; Lucía Spangenberg; Mailen Arleo; Mariana Brandes; Marilda Mendonça Siqueira; Marilda Tereza Mar da Rosa; María José Benitez-Galeano; Martín Graña; Matías Castells; Matías Victoria; Matías Salvo; Natalia Rego; Natalia Reyes; Pablo Smircich; Paola Cristina Resende; Rodney Colina; Tamara Fernandez-Calero; Tania Possi; Tatiana Schäffer Gregianini; Verónica Noya; Yasser Vega
EPI_ISL_750165	CENUR Litoral Norte – UdelaR, Salto, Uruguay.	Institut Pasteur de Montevideo	Ana Carolina Mendonça; Andrés Lizasoain; Camila Simoes; Cecilia Alonso; Cecilia Salazar; Daiana Mir; Fernando López-Tort; Fernando Motta; Gonzalo Bello; Ighor Arantes; Ignacio Ferrés; Jose Sotelo; Leticia Maya; Letícia Garay Martins; Luciana Appolinario; Lucía Spangenberg; Mailen Arleo; Mariana Brandes; Marilda Mendonça Siqueira; Marilda Tereza Mar da Rosa; María José Benitez-Galeano; Martín Graña; Matías Castells; Matías Victoria; Matías Salvo; Natalia Rego; Natalia Reyes; Pablo Smircich; Paola Cristina Resende; Rodney Colina; Tamara Fernandez-Calero; Tania Possi; Tatiana Schäffer Gregianini; Verónica Noya; Yasser Vega
EPI_ISL_2754313, EPI_ISL_2754314, EPI_ISL_2754315, EPI_ISL_2754316, EPI_ISL_2754317, EPI_ISL_2754318, EPI_ISL_2754370, EPI_ISL_2754398, EPI_ISL_2754468, EPI_ISL_2754517, EPI_ISL_2754549, EPI_ISL_2754581, EPI_ISL_2754608, EPI_ISL_2754628, EPI_ISL_2754665, EPI_ISL_2754806, EPI_ISL_2754825, EPI_ISL_2754843, EPI_ISL_2754854, EPI_ISL_2754856, EPI_ISL_2754952, EPI_ISL_2754957, EPI_ISL_2754958, EPI_ISL_2754960, EPI_ISL_2755057, EPI_ISL_2755084, EPI_ISL_2755120, EPI_ISL_2755494, EPI_ISL_2755598, EPI_ISL_2755733, EPI_ISL_2755857, EPI_ISL_2757850, EPI_ISL_2757851, EPI_ISL_2757852, EPI_ISL_2757853, EPI_ISL_2757854, EPI_ISL_2757855, EPI_ISL_2757856, EPI_ISL_2757857, EPI_ISL_2757858, EPI_ISL_2757859			
see above	CURE	Institut Pasteur de Montevideo	27000 Rocha; 9; Andres Lizasoain; Belén González; Cecilia Alonso; Daiana Mir; Departamento de Rocha; Emiliano Pereira; Gonzalo Bello; Ighor Arantes; Juan Zanetti; Lucia Bilbao; Luciana Griffero; Lucía Spangenberg; Mailen Arleo; Mariana Brandes; María José Benítez-Galeano; Matías Castells; Matías Salvo; Matías Victoria; Mauricio Méndez; Melissa Duquía; Natalia Rego; Natalia Reyes; Odhille Chappos; Pablo Smircich; Pía Techera; Rodney Colina; Tamara Fernández-Calero; Tania Possi; Verónica Noya
EPI_ISL_2031707, EPI_ISL_2031708, EPI_ISL_2031709, EPI_ISL_2031710, EPI_ISL_2031711, EPI_ISL_2031712, EPI_ISL_2031713, EPI_ISL_2031714, EPI_ISL_2031715, EPI_ISL_2031716, EPI_ISL_2031717, EPI_ISL_2031718, EPI_ISL_2031719, EPI_ISL_2031720, EPI_ISL_2031721, EPI_ISL_2031722, EPI_ISL_2031723, EPI_ISL_2031724, EPI_ISL_2031725, EPI_ISL_2031726, EPI_ISL_2031727, EPI_ISL_2031728, EPI_ISL_2031729, EPI_ISL_2031730, EPI_ISL_2031731, EPI_ISL_2031732, EPI_ISL_2031733, EPI_ISL_2031734, EPI_ISL_2031735, EPI_ISL_2031736, EPI_ISL_2031737, EPI_ISL_2031738, EPI_ISL_2031739, EPI_ISL_2031740, EPI_ISL_2031741, EPI_ISL_2031742, EPI_ISL_2031743, EPI_ISL_2031744, EPI_ISL_2031745, EPI_ISL_2031746, EPI_ISL_2031747, EPI_ISL_2031748, EPI_ISL_2031749, EPI_ISL_2031750, EPI_ISL_2031751, EPI_ISL_2031752, EPI_ISL_2031753, EPI_ISL_2031754, EPI_ISL_2031755, EPI_ISL_2031756, EPI_ISL_2031757, EPI_ISL_2031758, EPI_ISL_2031759, EPI_ISL_2031760, EPI_ISL_2031761, EPI_ISL_2031762			
see above	Centro de Innovación en Vigilancia Epidemiológica (CiVE), Institut Pasteur Montevideo, Uruguay	Centro de Innovación en Vigilancia Epidemiológica (CiVE), Institut Pasteur Montevideo, Uruguay	Alicia Costábile; Alvaro Fajardo; Andrés Lizosaín; Belén González; Bernardina Rivera; Cecilia Alonso; Cecilia Salazar; Gonzalo Moratorio; Gregorio Iraola; Henry Albornoz; Ignacio Ferrés; Inés Bellini; Juan Zanetti; Julio Medina; Lucia Bilbao; Luciana Griffero; Lucía Spangenberg; Ma Noel Bentancor; Ma Pía Techera; Mailen Arleo; Martina Alonso; María José Beníez; Matías Maidana; Mauricio Méndez; Melissa Duquía; Mercedes Paz; Natalia Rego; Natalia Reyes; Odhille Chappos; Paula Perbolianachis; Pilar Moreno; Rodney Colina; Rodrigo Arce; Tamara Fernández; Tania Possi
EPI_ISL_444493	Departamento de Laboratorios de Salud Publica (DLSP, Division Epidemiologia, Ministerio de Salud Publica)	Facultad de Ciencias (Sección Genética Evolutiva, Sección Virología).	Arbiza; Calleros, L.; Chiparelli, H.; Coppola, L.; Delfraro, A.; Frabasile, S.; Fuques, E.; Goni, N.; Grecco, S.; J. and Perez, R.; Panzera, Y.; Ramos, N.; Ramos, V.; Techera, C.
EPI_ISL_3696840, EPI_ISL_3696841, EPI_ISL_3696842, EPI_ISL_3696843	Genetica y Virologia (FC), Facultad de Ciencias & DLSP	Genetica y Virologia (FC), Facultad de Ciencias & DLSP	Arbiza, J.; Calleros, L.; Chiparelli, H.; Condon, E.; Coppola, L.; Cortinas; Delfraro, A.; Frabasile, S.; Goni, N.; Grecco, S.; M.N.; Marandino, A.; Mogdasy, C.; Panzera, Y.; Perez, R.; Ramas, V.; Ramos, N.; Sorhouet, C.; Techera, C.; Tomas, G.
EPI_ISL_936380, EPI_ISL_936381, EPI_ISL_936382, EPI_ISL_936383, EPI_ISL_936384, EPI_ISL_936385, EPI_ISL_936386			
see above	Genetica y Virologia, Facultad de Ciencias	Genetica y Virologia, Facultad de Ciencias	Arbiza, J.; Calleros, L.; Chiparelli, H.; Coppola, L.; Delfraro, A.; Frabasile, S.; Fuques, E.; Goni, N.; Grecco, S.; Marandino, A.; Mogdasy, C.; Panzera, Y.; Perez, R.; Ramas, V.; Ramos, N.; Sorhouet, C.; Techera, C.; Tomas, G.
EPI_ISL_2963518, EPI_ISL_2964686, EPI_ISL_2964705, EPI_ISL_2965557, EPI_ISL_2965558, EPI_ISL_2965559, EPI_ISL_2965560, EPI_ISL_2965561, EPI_ISL_2965562, EPI_ISL_2965563, EPI_ISL_2965564, EPI_ISL_2965565, EPI_ISL_2965568, EPI_ISL_2965569, EPI_ISL_2965570, EPI_ISL_2965571, EPI_ISL_2965581, EPI_ISL_2965583, EPI_ISL_2965584, EPI_ISL_2965585, EPI_ISL_2965586, EPI_ISL_3089994, EPI_ISL_3089995, EPI_ISL_3089996			
see above	Hospital Español	Centro de Innovación en Vigilancia Epidemiológica (CiVE), Institut Pasteur Montevideo, Uruguay	Alicia Costábile; Alvaro Fajardo; Ana Moller; Andrés Lizasoain; Belén González; Bernardina Rivera; Cecilia Alonso; Cecilia Salazar; Gonzalo Bello; Gonzalo Moratorio; Gregorio Iraola; Henry Albornoz; Ignacio Ferrés; Javier Hurtado; Juan Zanetti; Julio Medina; Luciana Griffero; Lucía Spangenberg; Ma Noel Bentancor; Ma Pía Techera; Mailen Arleo; Martina Alonso; Matías Maidana; Mauricio Méndez; Melissa Duquía; Mercedes Paz; Natalia Rego; Natalia Reyes; Nicolas Nin; Odhille Chappos; Paula Perbolianachis; Pilar Moreno; Rodney Colina; Rodrigo Arce; Tamara Fernández-Calero; Tania Possi; Veronica Noya; Viviana Bortagaray
EPI_ISL_1991974, EPI_ISL_1992100, EPI_ISL_1992241	Institut Pasteur de Montevideo	Institut Pasteur de Montevideo	Andrés Lizasoain; Camila Simoes; Cecilia Alonso; Daiana Mir; Fernando López-Tort; Gonzalo Bello; Jose Sotelo Silveira; Leticia Maya; Lucia Bilbao; Lucía Spangenberg; Mailen Arleo; Mariana Brandes; María José Benitez-Galeano; Martín Graña; Matías Castells; Matías Victoria; Matías Salvo; Natalia Rego; Natalia Reyes; Pablo Smircich; Rodney Colina; Tamara Fernandez-Calero; Tania Possi; Verónica Noya; Yasser Vega
EPI_ISL_747615, EPI_ISL_748138, EPI_ISL_748139, EPI_ISL_748140, EPI_ISL_748141, EPI_ISL_748142, EPI_ISL_748143, EPI_ISL_748144, EPI_ISL_748145			
see above	Instituto de Investigaciones Biológicas Clemente Estable	Institut Pasteur de Montevideo	Ana Carolina Mendonça; Andrés Lizasoain; Camila Simoes; Cecilia Alonso; Cecilia Salazar; Daiana Mir; Fernando López-Tort; Fernando Motta; Gonzalo Bello; Ighor Arantes; Ignacio Ferrés; Jose Sotelo; Leticia Maya; Letícia Garay Martins; Luciana Appolinario; Lucía Spangenberg; Mailen Arleo; Mariana Brandes; Marilda Mendonça Siqueira; Marilda Tereza Mar da Rosa; María José Benitez-Galeano; Martín Graña; Matías Castells; Matías Victoria; Matías Salvo; Natalia Rego; Natalia Reyes; Pablo Smircich; Paola Cristina Resende; Rodney Colina; Tamara Fernandez-Calero; Tania Possi; Tatiana Schäffer Gregianini; Verónica Noya; Yasser Vega
EPI_ISL_2964381, EPI_ISL_2964383	Laboratorio DILAVE/MGAP-INIA-UdelaR - Tacuarembó	Centro de Innovación en Vigilancia Epidemiológica (CiVE), Institut Pasteur Montevideo, Uruguay	Alicia Costábile; Alvaro Fajardo; Ana Moller; Andrés Lizasoain; Belén González; Bernardina Rivera; Cecilia Alonso; Cecilia Salazar; Gonzalo Bello; Gonzalo Moratorio; Gregorio Iraola; Henry Albornoz; Ignacio Ferrés; Javier Hurtado; Juan Zanetti; Julio Medina; Luciana Griffero; Lucía Spangenberg; Ma Noel Bentancor; Ma Pía Techera; Mailen Arleo; Martina Alonso; Matías Maidana; Mauricio Méndez; Melissa Duquía; Mercedes Paz; Natalia Rego; Natalia Reyes; Nicolas Nin; Odhille Chappos; Paula Perbolianachis; Pilar Moreno; Rodney Colina; Rodrigo Arce; Tamara Fernández-Calero; Tania Possi; Veronica Noya; Viviana Bortagaray
EPI_ISL_750163, EPI_ISL_750164, EPI_ISL_751201	Laboratorio DILAVE/MGAP-INIA-UdelaR - Tacuarembó	Institut Pasteur de Montevideo	Ana Carolina Mendonça; Andrés Lizasoain; Camila Simoes; Cecilia Alonso; Cecilia Salazar; Daiana Mir; Fernando López-Tort; Fernando Motta; Gonzalo Bello; Ighor Arantes; Ignacio Ferrés; Jose Sotelo; Leticia Maya; Letícia Garay Martins; Luciana Appolinario; Lucía Spangenberg; Mailen Arleo; Mariana Brandes; Marilda Mendonça Siqueira; Marilda Tereza Mar da Rosa; María José Benitez-Galeano; Martín Graña; Matías Castells; Matías Victoria; Matías Salvo; Natalia Rego; Natalia Reyes; Pablo Smircich; Paola Cristina Resende; Rodney Colina; Tamara Fernandez-Calero; Tania Possi; Tatiana Schäffer Gregianini; Verónica Noya; Yasser Vega
EPI_ISL_457940, EPI_ISL_457941, EPI_ISL_457942, EPI_ISL_457943, EPI_ISL_457944, EPI_ISL_457945, EPI_ISL_457946, EPI_ISL_457947, EPI_ISL_457948, EPI_ISL_457949, EPI_ISL_457950, EPI_ISL_457951, EPI_ISL_457952, EPI_ISL_457953, EPI_ISL_457954, EPI_ISL_457955, EPI_ISL_457956, EPI_ISL_457957, EPI_ISL_457958, EPI_ISL_457959, EPI_ISL_457960, EPI_ISL_457961, EPI_ISL_457962, EPI_ISL_457963, EPI_ISL_457964, EPI_ISL_457965, EPI_ISL_457966, EPI_ISL_457967, EPI_ISL_457968, EPI_ISL_457969, EPI_ISL_457970, EPI_ISL_457971, EPI_ISL_457972, EPI_ISL_457973, EPI_ISL_480428, EPI_ISL_480429, EPI_ISL_480431, EPI_ISL_480432, EPI_ISL_480433, EPI_ISL_480434, EPI_ISL_480435, EPI_ISL_480436, EPI_ISL_480437, EPI_ISL_480438			
see above	Laboratorio de Biología Molecular Asociación Española Primera en Salud	Departments of Pathology and Medicine, New York University School of Medicine	Adriana Heguy; Christian Marier; Gael Westby; Gonzalo Manrique; Maria Noel Zubillaga; Maria Victoria Elizondo; Matthew T Maurano; Paul Zappile
EPI_ISL_2427504, EPI_ISL_2427505, EPI_ISL_2427506, EPI_ISL_2427507, EPI_ISL_2427508, EPI_ISL_2427509, EPI_ISL_2427510, EPI_ISL_2427511, EPI_ISL_2427512, EPI_ISL_2427513, EPI_ISL_2427514, EPI_ISL_2427515, EPI_ISL_2427516, EPI_ISL_2427517, EPI_ISL_2427518, EPI_ISL_2427519, EPI_ISL_2427520, EPI_ISL_2427521, EPI_ISL_2427522, EPI_ISL_2427523, EPI_ISL_2427524, EPI_ISL_2427525, EPI_ISL_2427526, EPI_ISL_2427527, EPI_ISL_2427528, EPI_ISL_2427529, EPI_ISL_2427530, EPI_ISL_2427531, EPI_ISL_2427532, EPI_ISL_2427533, EPI_ISL_2427534, EPI_ISL_2427535, EPI_ISL_2427536, EPI_ISL_2427537, EPI_ISL_2427538, EPI_ISL_2427539, EPI_ISL_2427540, EPI_ISL_2427541, EPI_ISL_2427542, EPI_ISL_2427543, EPI_ISL_2427544, EPI_ISL_2427545, EPI_ISL_2427546, EPI_ISL_2427547, EPI_ISL_2427548, EPI_ISL_2427549, EPI_ISL_2427550, EPI_ISL_2427551, EPI_ISL_2427552, EPI_ISL_2427553, EPI_ISL_2427554, EPI_ISL_2427555, EPI_ISL_2427556, EPI_ISL_2427557, EPI_ISL_2427558, EPI_ISL_2427559, EPI_ISL_2427560, EPI_ISL_2427561, EPI_ISL_2427562, EPI_ISL_2427563, EPI_ISL_2427564, EPI_ISL_2427565, EPI_ISL_2427566, EPI_ISL_2427567, EPI_ISL_2427568, EPI_ISL_2427569, EPI_ISL_2427570, EPI_ISL_2427571, EPI_ISL_2427572, EPI_ISL_2427573, EPI_ISL_2427574, EPI_ISL_2427575, EPI_ISL_2427576, EPI_ISL_2427577, EPI_ISL_2427578, EPI_ISL_2427579, EPI_ISL_2427580, EPI_ISL_2427581, EPI_ISL_2427582, EPI_ISL_2427583, EPI_ISL_2427584, EPI_ISL_2427585, EPI_ISL_2427586, EPI_ISL_2427587, EPI_ISL_2427588, EPI_ISL_2427589, EPI_ISL_2427590, EPI_ISL_2427591, EPI_ISL_2427592, EPI_ISL_2427593, EPI_ISL_2427594, EPI_ISL_2427595, EPI_ISL_2427596, EPI_ISL_2427597, EPI_ISL_2427598, EPI_ISL_2427599, EPI_ISL_2427600, EPI_ISL_2427601, EPI_ISL_2427602, EPI_ISL_2427603, EPI_ISL_2427604, EPI_ISL_2427605, EPI_ISL_2427606, EPI_ISL_2427607, EPI_ISL_2427608, EPI_ISL_2427609, EPI_ISL_2427610, EPI_ISL_2427611, EPI_ISL_2427612, EPI_ISL_2427613, EPI_ISL_2427614, EPI_ISL_2427615, EPI_ISL_2427616, EPI_ISL_2427617, EPI_ISL_2427618, EPI_ISL_2427619, EPI_ISL_2427620, EPI_ISL_2427621, EPI_ISL_2427622, EPI_ISL_2427623, EPI_ISL_2427624, EPI_ISL_2427625, EPI_ISL_2427626, EPI_ISL_2427627, EPI_ISL_2427628, EPI_ISL_2427629, EPI_ISL_2427630, EPI_ISL_2427631, EPI_ISL_2427632, EPI_ISL_2427633, EPI_ISL_2427634, EPI_ISL_2427635, EPI_ISL_2427636, EPI_ISL_2427637, EPI_ISL_2427638, EPI_ISL_2427639, EPI_ISL_2427640, EPI_ISL_2427641, EPI_ISL_2427642, EPI_ISL_2427643, EPI_ISL_2427644, EPI_ISL_2427645, EPI_ISL_2427646, EPI_ISL_2427647, EPI_ISL_2427648, EPI_ISL_2427649, EPI_ISL_2427650, EPI_ISL_2427651, EPI_ISL_2427652, EPI_ISL_2427653, EPI_ISL_2427654, EPI_ISL_2427655, EPI_ISL_2427656, EPI_ISL_2427657, EPI_ISL_2427658, EPI_ISL_2427659, EPI_ISL_2427660, EPI_ISL_2427661, EPI_ISL_2427662, EPI_ISL_2427663, EPI_ISL_2427664, EPI_ISL_2427665, EPI_ISL_2427666, EPI_ISL_2427667, EPI_ISL_2427668, EPI_ISL_2427669, EPI_ISL_2427670, EPI_ISL_2427671, EPI_ISL_2427672, EPI_ISL_2427673, EPI_ISL_2427674, EPI_ISL_2427675, EPI_ISL_2427676, EPI_ISL_2427677, EPI_ISL_2427678, EPI_ISL_2427679, EPI_ISL_2427680, EPI_ISL_2427681, EPI_ISL_2427682, EPI_ISL_2427683, EPI_ISL_2427684, EPI_ISL_2427685, EPI_ISL_2427686, EPI_ISL_2427687, EPI_ISL_2427688, EPI_ISL_2427689, EPI_ISL_2427690, EPI_ISL_2427691, EPI_ISL_2427692, EPI_ISL_2427693, EPI_ISL_2427694, EPI_ISL_2427695, EPI_ISL_2427696, EPI_ISL_2427697, EPI_ISL_2427698, EPI_ISL_2427699, EPI_ISL_2427700, EPI_ISL_2427701, EPI_ISL_2427702, EPI_ISL_2427703, EPI_ISL_2427704, EPI_ISL_2427705, EPI_ISL_2427706, EPI_ISL_2427707, EPI_ISL_2427708, EPI_ISL_2427709, EPI_ISL_2427710, EPI_ISL_2427711, EPI_ISL_2427712, EPI_ISL_2427713, EPI_ISL_2427714, EPI_ISL_2427715, EPI_ISL_2427716, EPI_ISL_2427717, EPI_ISL_2427718, EPI_ISL_2427719, EPI_ISL_2427720, EPI_ISL_2427721, EPI_ISL_2427722, EPI_ISL_2427723, EPI_ISL_2427724, EPI_ISL_2427725, EPI_ISL_2427726, EPI_ISL_2427727, EPI_ISL_2427728, EPI_ISL_2427729, EPI_ISL_2427730, EPI_ISL_2427731, EPI_ISL_2427732, EPI_ISL_2427733, EPI_ISL_2427734, EPI_ISL_2427735, EPI_ISL_2427736, EPI_ISL_2427737, EPI_ISL_2427738, EPI_ISL_2427739, EPI_ISL_2427740, EPI_ISL_2427741, EPI_ISL_2427742, EPI_ISL_2427743, EPI_ISL_2427744, EPI_ISL_2427745, EPI_ISL_2427746, EPI_ISL_2427747, EPI_ISL_2427748, EPI_ISL_2427749, EPI_ISL_2427750, EPI_ISL_2427751, EPI_ISL_2427752, EPI_ISL_2427753, EPI_ISL_2427754, EPI_ISL_2427755, EPI_ISL_2427756, EPI_ISL_2427757, EPI_ISL_2427758, EPI_ISL_2427759, EPI_ISL_2427760, EPI_ISL_2427761, EPI_ISL_2427762, EPI_ISL_2427763, EPI_ISL_2427764, EPI_ISL_2427765, EPI_ISL_2427766, EPI_ISL_2427767, EPI_ISL_2427768, EPI_ISL_2427769, EPI_ISL_2427770, EPI_ISL_2427771, EPI_ISL_2427772, EPI_ISL_2427773, EPI_ISL_2427774, EPI_ISL_2427775, EPI_ISL_2427776, EPI_ISL_2427777, EPI_ISL_2427778, EPI_ISL_2427779			
see above	Laboratorio de Biología Molecular Médica Uruguaya	Departments of Pathology and Medicine, New York University School of Medicine	Adriana Heguy; Cecilia Sorhouet; Christian Marier; Dacia Dimartino; Gonzalo Manrique; Maria Cristina Mogdasy; Maria Noel Zubillaga; Maria Victoria Elizondo; Paul Zappile
EPI_ISL_2963634, EPI_ISL_2964223, EPI_ISL_2964382, EPI_ISL_2964384, EPI_ISL_2964385, EPI_ISL_2964387, EPI_ISL_2964388, EPI_ISL_2964483, EPI_ISL_2964558, EPI_ISL_2964559, EPI_ISL_2964561, EPI_ISL_2964562, EPI_ISL_2964563, EPI_ISL_2964567, EPI_ISL_2964568, EPI_ISL_2964570, EPI_ISL_2964571, EPI_ISL_2964587, EPI_ISL_2964589, EPI_ISL_2964590, EPI_ISL_2964591, EPI_ISL_2964592, EPI_ISL_2964593, EPI_ISL_2964594, EPI_ISL_2964596, EPI_ISL_2964597, EPI_ISL_2964625, EPI_ISL_2964626, EPI_ISL_2964633, EPI_ISL_2964634, EPI_ISL_2964635, EPI_ISL_2964638, EPI_ISL_2964651, EPI_ISL_2964653, EPI_ISL_2964654, EPI_ISL_2964655, EPI_ISL_2964658, EPI_ISL_2964659, EPI_ISL_2964660, EPI_ISL_2964671, EPI_ISL_2964672, EPI_ISL_2964675, EPI_ISL_2964676, EPI_ISL_2964678, EPI_ISL_2964679, EPI_ISL_2964680, EPI_ISL_2964682, EPI_ISL_2964683, EPI_ISL_2964691, EPI_ISL_2964695, EPI_ISL_2965573, EPI_ISL_2965574, EPI_ISL_2965576, EPI_ISL_2965578			
see above	Laboratorio de Biología Molecular del Sanatorio Americano	Centro de Innovación en Vigilancia Epidemiológica (CiVE), Institut Pasteur Montevideo, Uruguay	Alicia Costábile; Alvaro Fajardo; Ana Moller; Andrés Lizasoain; Belén González; Bernardina Rivera; Cecilia Alonso; Cecilia Salazar; Gonzalo Bello; Gonzalo Moratorio; Gregorio Iraola; Henry Albornoz; Ignacio Ferrés; Javier Hurtado; Juan Zanetti; Julio Medina; Luciana Griffero; Lucía Spangenberg; Ma Noel Bentancor; Ma Pía Techera; Mailen Arleo; Martina Alonso; Matías Maidana; Mauricio Méndez; Melissa Duquía; Mercedes Paz; Natalia Rego; Natalia Reyes; Nicolas Nin; Odhille Chappos; Paula Perbolianachis; Pilar Moreno; Rodney Colina; Rodrigo Arce; Tamara Fernández-Calero; Tania Possi; Veronica Noya; Viviana Bortagaray
EPI_ISL_2964557, EPI_ISL_2964560, EPI_ISL_2964564, EPI_ISL_2964565, EPI_ISL_2964566, EPI_ISL_2964569, EPI_ISL_2964588, EPI_ISL_2964595, EPI_ISL_2964636, EPI_ISL_2964637, EPI_ISL_2964646, EPI_ISL_2964648, EPI_ISL_2964649, EPI_ISL_2964650, EPI_ISL_2964656, EPI_ISL_2964657, EPI_ISL_2964674, EPI_ISL_2965575, EPI_ISL_2965579, EPI_ISL_2965580, EPI_ISL_3089997, EPI_ISL_3089998, EPI_ISL_3089999, EPI_ISL_3090000, EPI_ISL_3090001, EPI_ISL_3090002, EPI_ISL_3090003, EPI_ISL_3090004, EPI_ISL_3090005, EPI_ISL_3090006, EPI_ISL_3090007, EPI_ISL_3090008, EPI_ISL_3090009, EPI_ISL_3090010, EPI_ISL_3090011, EPI_ISL_3090012, EPI_ISL_3090013, EPI_ISL_3090014, EPI_ISL_3090015, EPI_ISL_3090016, EPI_ISL_3090017, EPI_ISL_3090018, EPI_ISL_3090019, EPI_ISL_3090020, EPI_ISL_3090021, EPI_ISL_3090022, EPI_ISL_3090023, EPI_ISL_3090024, EPI_ISL_3090025, EPI_ISL_3090026, EPI_ISL_3090027, EPI_ISL_3090028, EPI_ISL_3090029, EPI_ISL_3090030, EPI_ISL_3090031, EPI_ISL_3090032, EPI_ISL_3090033, EPI_ISL_3090034, EPI_ISL_3090035, EPI_ISL_3090036, EPI_ISL_3090037			
see above	Laboratorio de Diagnóstico Molecular, Centro de Innovación en Vigilancia Epidemiológica (CiVE), Institut Pasteur Montevideo, Uruguay	Centro de Innovación en Vigilancia Epidemiológica (CiVE), Institut Pasteur Montevideo, Uruguay	Alicia Costábile; Alvaro Fajardo; Ana Moller; Andrés Lizasoain; Belén González; Bernardina Rivera; Cecilia Alonso; Cecilia Salazar; Gonzalo Bello; Gonzalo Moratorio; Gregorio Iraola; Henry Albornoz; Ignacio Ferrés; Javier Hurtado; Juan Zanetti; Julio Medina; Luciana Griffero; Lucía Spangenberg; Ma Noel Bentancor; Ma Pía Techera; Mailen Arleo; Martina Alonso; Matías Maidana; Mauricio Méndez; Melissa Duquía; Mercedes Paz; Natalia Rego; Natalia Reyes; Nicolas Nin; Odhille Chappos; Paula Perbolianachis; Pilar Moreno; Rodney Colina; Rodrigo Arce; Tamara Fernández-Calero; Tania Possi; Veronica Noya; Viviana Bortagaray

EPI_ISL_2964687, EPI_ISL_2964689, EPI_ISL_2964690, EPI_ISL_3098718	Laboratorio de Diagnóstico Molecular, Centro de Innovación en Vigilancia Epidemiológica (CiVE), Institut Pasteur Montevideo, Uruguay	Institut Pasteur de Montevideo	Alicia Costábile; Alvaro Fajardo; Ana Moller; Andrés Lizasoain; Belén González; Bernardina Rivera; Cecilia Alonso; Cecilia Salazar; Gonzalo Bello; Gonzalo Moratorio; Gregorio Iraola; Henry Albornoz; Ignacio Ferrés; Javier Hurtado; Juan Zanetti; Julio Medina; Luciana Griffero; Lucía Spangenberg; Ma Noel Bentancor; Ma Pía Techera; Mailen Arleo; Martina Alonso; Matías Maidana; Mauricio Méndez; Melissa Duquía; Mercedes Paz; Natalia Rego; Natalia Reyes; Nicolas Nin; Odhille Chappos; Paula Perbolianachis; Pilar Moreno; Rodney Colina; Rodrigo Arce; Tamara Fernández-Calero; Tania Possi; Veronica Noya; Viviana Bortagaray
EPI_ISL_2963519, EPI_ISL_2964004, EPI_ISL_2964380, EPI_ISL_2964386, EPI_ISL_2964693, EPI_ISL_2964694, EPI_ISL_2964696, EPI_ISL_2964698, EPI_ISL_2964699, EPI_ISL_2964701, EPI_ISL_2964702, EPI_ISL_2964703, EPI_ISL_2965572, EPI_ISL_3089975, EPI_ISL_3089976, EPI_ISL_3089977, EPI_ISL_3089978, EPI_ISL_3089979, EPI_ISL_3089980, EPI_ISL_3089981, EPI_ISL_3089982, EPI_ISL_3089983, EPI_ISL_3089984, EPI_ISL_3089985, EPI_ISL_3089986, EPI_ISL_3089987, EPI_ISL_3089988, EPI_ISL_3089989, EPI_ISL_3089990, EPI_ISL_3089991, EPI_ISL_3089992, EPI_ISL_3089993, EPI_ISL_3090038, EPI_ISL_3090039, EPI_ISL_3090040			
see above	Laboratorio de Virología Molecular, Centro Universitario Regional del Litoral Norte. Universidad de la República, Salto, Uruguay	Centro de Innovación en Vigilancia Epidemiológica (CiVE), Institut Pasteur Montevideo, Uruguay	Alicia Costábile; Alvaro Fajardo; Ana Moller; Andrés Lizasoain; Belén González; Bernardina Rivera; Cecilia Alonso; Cecilia Salazar; Gonzalo Bello; Gonzalo Moratorio; Gregorio Iraola; Henry Albornoz; Ignacio Ferrés; Javier Hurtado; Juan Zanetti; Julio Medina; Luciana Griffero; Lucía Spangenberg; Ma Noel Bentancor; Ma Pía Techera; Mailen Arleo; Martina Alonso; Matías Maidana; Mauricio Méndez; Melissa Duquía; Mercedes Paz; Natalia Rego; Natalia Reyes; Nicolas Nin; Odhille Chappos; Paula Perbolianachis; Pilar Moreno; Rodney Colina; Rodrigo Arce; Tamara Fernández-Calero; Tania Possi; Veronica Noya; Viviana Bortagaray
EPI_ISL_426583	Microbial Genomics Laboratory, Institut Pasteur Monteivdeo	Microbial Genomics Laboratory, Institut Pasteur Montevideo, Uruguay	Cecilia Salazar; Florencia Díaz-Viraqué; Gonzalo Moratorio; Gregorio Iraola; Marianoel Pereira; Pilar Moreno
EPI_ISL_426479, EPI_ISL_426480, EPI_ISL_429257	Microbial Genomics Laboratory, Institut Pasteur Montevideo	Microbial Genomics Laboratory, Institut Pasteur Montevideo	Cecilia Salazar; Florencia Díaz-Viraqué; Gonzalo Moratorio; Gregorio Iraola; Marianoel Pereira; Pilar Moreno
EPI_ISL_426476, EPI_ISL_426477, EPI_ISL_426478	Microbial Genomics Laboratory, Institut Pasteur Montevideo	Microbial Genomics Laboratory, Institut Pasteur Montevideo, Uruguay	Cecilia Salazar; Florencia Díaz-Viraqué; Gonzalo Moratorio; Gregorio Iraola; Marianoel Pereira; Pilar Moreno
EPI_ISL_426481, EPI_ISL_426482	Microbial Genomics Laboratory, Institut Pasteur Montevideo, Uruguay	Microbial Genomics Laboratory, Institut Pasteur Montevideo	Cecilia Salazar; Florencia Díaz-Viraqué; Gonzalo Moratorio; Gregorio Iraola; Marianoel Pereira; Pilar Moreno
EPI_ISL_426584	Microbial Genomics Laboratory, Institut Pasteur Montevideo, Uruguay	Microbial Genomics Laboratory, Institut Pasteur Montevideo, Uruguay	Cecilia Salazar; Florencia Díaz-Viraqué; Gonzalo Moratorio; Gregorio Iraola; Marianoel Pereira; Pilar Moreno
EPI_ISL_480331, EPI_ISL_480332, EPI_ISL_480333, EPI_ISL_480334, EPI_ISL_480335, EPI_ISL_480336, EPI_ISL_480337, EPI_ISL_480338, EPI_ISL_480339, EPI_ISL_480340, EPI_ISL_480341, EPI_ISL_480342, EPI_ISL_480343, EPI_ISL_480344, EPI_ISL_480345, EPI_ISL_480346, EPI_ISL_480347, EPI_ISL_480348			
see above	Microbial Genomics Laboratory, Institut Pasteur de Montevideo	Microbial Genomics Laboratory, Institut Pasteur de Montevideo	Cecilia Salazar; Gonzalo Moratorio; Gregorio Iraola; Ignacio Ferrés; Marianoel Pereira; Pilar Moreno
EPI_ISL_540430	Microbial Genomics Laboratory, Institut Pasteur de Montevideo, Montevideo, Uruguay	Microbial Genomics Laboratory, Institut Pasteur de Montevideo, Montevideo, Uruguay	Cecilia Salazar; Gonzalo Moratorio; Gregorio Iraola; Marianoel Pereira; Pilar Moreno
EPI_ISL_748667, EPI_ISL_749036, EPI_ISL_749148, EPI_ISL_749149, EPI_ISL_749150, EPI_ISL_749151, EPI_ISL_749152, EPI_ISL_749153, EPI_ISL_749154, EPI_ISL_749155, EPI_ISL_749238, EPI_ISL_749474, EPI_ISL_749706, EPI_ISL_749906, EPI_ISL_750108, EPI_ISL_750161, EPI_ISL_750162, EPI_ISL_750166, EPI_ISL_750168, EPI_ISL_750169, EPI_ISL_750170, EPI_ISL_750171, EPI_ISL_750172, EPI_ISL_750173, EPI_ISL_750174, EPI_ISL_750176, EPI_ISL_750177, EPI_ISL_750178, EPI_ISL_750179, EPI_ISL_750256, EPI_ISL_750430, EPI_ISL_750820, EPI_ISL_751011, EPI_ISL_2752749, EPI_ISL_2753953, EPI_ISL_2753954, EPI_ISL_2753955, EPI_ISL_2753956, EPI_ISL_2753957, EPI_ISL_2753959, EPI_ISL_2753960, EPI_ISL_2753962, EPI_ISL_2753963, EPI_ISL_2753964, EPI_ISL_2753966, EPI_ISL_2753967, EPI_ISL_2753990, EPI_ISL_2754023, EPI_ISL_2754024, EPI_ISL_2754025, EPI_ISL_2754026, EPI_ISL_2754027, EPI_ISL_2754028, EPI_ISL_2754029, EPI_ISL_2754030, EPI_ISL_2754031, EPI_ISL_2754032, EPI_ISL_2754033, EPI_ISL_2754034, EPI_ISL_2754035, EPI_ISL_2754043, EPI_ISL_2754044, EPI_ISL_2754072, EPI_ISL_2754073, EPI_ISL_2754095, EPI_ISL_2754096, EPI_ISL_2754115, EPI_ISL_2754129, EPI_ISL_2754130, EPI_ISL_2754131, EPI_ISL_2754132, EPI_ISL_2754139, EPI_ISL_2754195, EPI_ISL_2754203, EPI_ISL_2754310, EPI_ISL_2754311, EPI_ISL_2754312, EPI_ISL_2768030			
see above	Sanatorio Americano	Institut Pasteur de Montevideo	Ana Carolina Mendonça; Andres Lizasoain; Andrés Lizasoain; Belén González; Camila Simoes; Cecilia Alonso; Cecilia Salazar; Daiana Mir; Emiliano Pereira; Fernando López-Tort; Fernando Motta; Gonzalo Bello; Ighor Arantes; Ignacio Ferrés; Jose Sotelo; Juan Zanetti; Leticia Maya; Letícia Garay Martins; Lucia Bilbao; Luciana Appolinario; Luciana Griffero; Lucía Spangenberg; Lucía Spangenberg; Mailen Arleo; Mariana Brandes; Marilda Mendonça Siqueira; Marilda Tereza Mar da Rosa; María José Benitez-Galeano; Martín Graña; María José Benítez-Galeano; Matías Castells; Matías Victoria; Matías Castells; Matías Salvo; Matías Victoria; Mauricio Méndez; Melissa Duquía; Natalia Rego; Natalia Reyes; Odhille Chappos; Pablo Smircich; Paola Cristina Resende; Pía Techera; Rodney Colina; Tamara Fernandez-Calero; Tamara Fernández-Calero; Tania Possi; Tatiana Schäffer Gregianini; Verónica Noya; Verónica Noya; Yasser Vega
